# Triplet‐drug chemotherapy combined with anti‐EGFR antibody as an effective therapy for patients with initially unresectable metastatic colorectal cancer: a meta-analysis

**DOI:** 10.1186/s12957-023-03256-7

**Published:** 2023-11-18

**Authors:** Muyou Tian, Huifen Li, Wenjing Dong, Yuhong Li, Ting Jiang, Yanhua Lv, Jianxiong Zeng, Xiaomei Jiang, Zhaofeng Yin, Jianjun Xiao

**Affiliations:** 1 Zhongshan City People’s Hospital, Affiliated Zhongshan Hospital of Sun Yat-Sen University, Zhongshan, 528400 Guangdong China; 2 State Key Laboratory of Oncology in South China, Collaborative Innovation Center for Cancer Medicine, Sun Yat-Sen University Cancer Center, Guangzhou, 510000 Guangdong China; 3 Department of Medical Oncology, Sun Yat-Sen University Cancer Center, Guangzhou, 510000 Guangdong China; 4 Xiangyang Central Hospital, Affiliated Hospital of Hubei University of Arts and Science, Xiangyang, 441000 Hubei China

**Keywords:** Unresectable metastatic colorectal cancer, Triplet, Drug chemotherapy, Anti, EGFR antibody

## Abstract

The meta-analysis aimed to assess the clinical efficacy of chemotherapeutic triplet‐drug regimen combined with anti‐EGFR antibody in patients with initially unresectable metastatic colorectal cancer (mCRC). A systematic literature search was performed in PubMed Publisher. Studies evaluating FOLFOXIRI combine with panitumumab or cetuximab as the therapy for initially unresectable mCRC were included. The primary outcome was objective response rate (ORR) and rate of R0 resections. The secondary outcomes included overall survival (OS), progression-free survival (PFS), and grades 3 or 4 adverse events. R software (version 4.0.2) and RevMan (version 5.3) were used to analyze the extracted data. The studies included were published between 2010 and 2021, involving four single-arm phase II trials and two randomized phase II trials. A total of 6 studies with 282 patients were included. The data showed a significant benefit for the FOLFOXIRI + anti-EGFR antibody arm compared with FOLFOXIRI arm (*RR* 1.33; 95% *CI*, 1.13–1.58; *I*^2^ = 0%, *P* < 0.05). The pooled ORR and pooled rate of R0 resection in patients who receiving FOLFOXIRI + anti-EGFR antibody were 85% (95% *CI*, 0.78–0.91; *I*^2^ = 58%) and 42% (95% *CI*, 0.32–0.53; *I*^2^ = 62%), respectively. The range of median PFS between all the six studies was 9.5–15.5 months, with weighted pooled median PFS mean 11.7 months. The range of median OS between all the four studies was 24.7–37 months, with weighted pooled median PFS mean 31.9 months. The common grades 3 and 4 adverse events were diarrhea and neutropenia. Our findings show that triplet-drug chemotherapy (FOLFOXIRI) combined with anti-EGFR antibody (panitumumab or cetuximab) represents a very effective therapeutic combination associated with a significant ORR and R0 rection rate for patients with molecularly unselected and surgically unresectable metastatic CRC.

## Introduction

Colorectal cancer (CRC) is one of the most common malignancies in the world and is the leading cause of cancer-related death [1]. More than half of patients will develop metastases during the course of disease [2]. Systemic chemotherapy is the main method to treat metastatic colorectal cancer (mCRC) [3]. However, the long-term outcome of patients with mCRC remains unfavorable unless a resection of metastatic disease can be performed [4]. Initially, non-resectable metastases have the possibility of achieving complete removal of all tumor masses after tumor shrinkage due to the effective chemotherapy regimens, emerging biological agents, and innovative surgical techniques (a process known as conversion therapy) [5]. Especially, it may significantly improve 5-year survival rate to 33% if achieved R0 resection [6]. As there is a correlation between objective response rate (ORR) and the resection rate, it might be important to develop more effective regimens that are able to induce tumor shrinkage [7].

The double FOLFOX or FOLFIRI regimen, which consists of the chemotherapeutic drugs fluorouracil, oxaliplatin, and irinotecan, is currently the most commonly used regimen for the treatment of colorectal cancer [8]. However, it is observed that a triplet regimen (FOLFOXIRI) with or without bevacizumab yields higher response, resection rates, and OS compared with doublet regimens among mCRC patients [9]. But quite a few adverse event occurs, especially diarrhea and neutropenia, leading to intolerance to the triplet regimen [10]. The incidence of adverse event may be reduced by reducing the dosage of drugs such as irinotecan [11]. Recently, the triple regimens plus an anti-EGFR monoclonal antibody (i.e., cetuximab or panitumumab) in some phase II trials demonstrated remarkable activity results, at the price of an increased rate of mucosal toxic effect (mainly diarrhea) [12]. Now there are some ongoing trials with triplet chemotherapy plus anti-EGFR in patients with mCRC [13]. Meta-analysis is to reflect the relevant research results in the existing literature more objectively and comprehensively, so as to obtain a more accurate understanding. Therefore, we performed a meta-analysis to assess the clinical efficacy of chemotherapeutic triplet‐drug regimen combined with anti‐EGFR antibody in patients with initially unresectable mCRC, including rate of surgical conversion and long-term outcomes.

## Materials and methods

### Literature search strategy

A systematic literature search was performed in PubMed from the inception to June 2021. English languages were searched. Retrieval keywords strategy included the following: ((FOLFOXIRI) OR (modified FOLFOXIRI) OR (fluorouracil irinotecan oxaliplatin)) AND ((colorectal cancer) OR (colon cancer) OR (rectal cancer)) AND ((panitumumab) OR (cetuximab) OR (antibody EGFR TKI inhibitor)).

### Inclusion and exclusion criteria

Only prospective clinical trials were included, regardless of the controlled group, which used triplet‐drug regimen combined with anti‐EGFR antibody for the conversion therapy of unresectable mCRC. All the following criteria had to be met for inclusion in the meta-analysis: (1) phases 2 or 3 trials involving patients with mCRC, (2) patients who were received to FOLFOXIRI + panitumumab or FOLFOXIRI + cetuximab regimens, and (3) available data that can be pooled. Studies were excluded if they met any of the following criteria: (i) repetitive publication, (ii) small sample size, (iii) abstract only, and (iv) no sufficient raw data and data unavailable on request.

### Data extraction

Based on the aforementioned strategies, studies were selected, and their eligibility was confirmed by two independent reviewers. The following information was extracted from each study: ORR, rate of R0 resections, rate of overall resections, median OS, median PFS, and the incidence of adverse events.

### Statistical analysis

Statistical analysis was performed using R software (version 4.0.2; the R Foundation, Vienna, Austria) for single-arm trials. For dichotomous variables (resections and objective responses), we calculated raw proportions of events divided by the total number of clinically evaluable patients. We calculated weighted pooled rates of events by random-effects model because of the heterogeneity in study size and to the large variations in proportions. Review Manager software (RevMan, version 5.3 for Windows; the Cochrane Collaboration, Oxford, UK) was used to conduct the meta-analysis for controlled trials. The ORR, the R0 resection rates, and incidence of adverse events were pooled through risk ratio (RR). The *χ*^2^ test was used to evaluate heterogeneity in the data. The fixed-effects model was used for studies without significant heterogeneity (*I*^2^ ≤ 50% or *P* ≥ 0.10), whereas the random-effects model was used for studies with significant heterogeneity. Median pooled-weighted OS and PFS were calculated with descriptive statistics. Due to the small number of included trials (< 10), we did not examine publication bias with Begg and Egger tests. The data obtained using Begg and Egger tests have poor results and cannot achieve the analysis purpose.

## Results

### Literature search and included studies

**Fig. 1:**
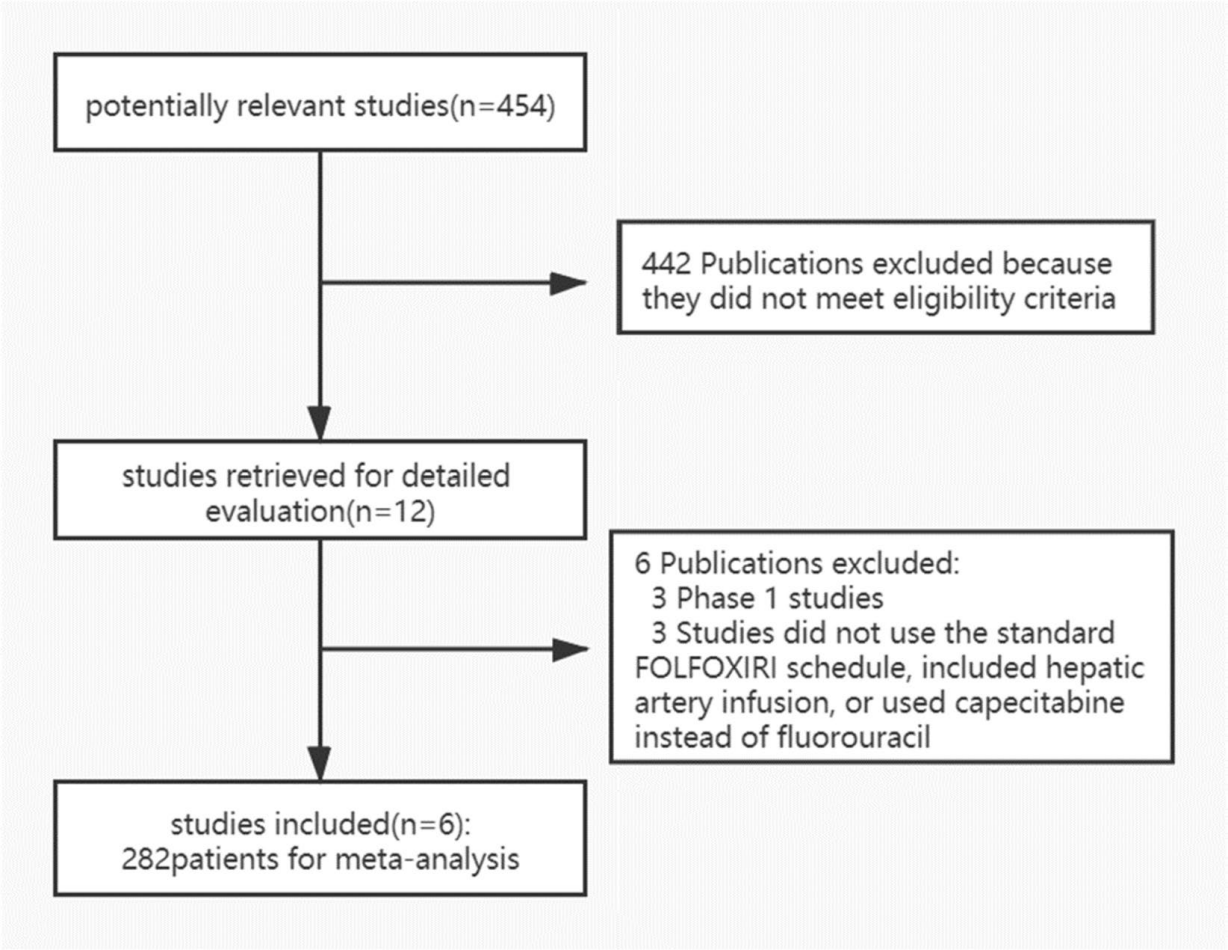
Flowchart of the included studies

A total of 454 potentially relevant papers were found according to the search strategy. The search term for literature collection is triplet dry chemistry (FOLFOXIRI). Four-hundred forty-two papers were excluded after screening the titles and abstracts. After that, 12 papers were selected for full-text assessment, of which 3 papers were excluded because of phase I studies, 3 papers were excluded because of not using the standard triplet‐drug chemotherapy (FOLFOXIRI), included hepatic artery infusion, or used capecitabine instead of fluorouracil. Finally, a total of 6 papers with 282 patients were included. The process of study selection is illustrated in Fig. 1.

### Study characteristics

The studies included were published between 2010 and 2021, involving four single-arm phase II trials and two randomized phase II trials [14]. Studies involving triplet‐drug chemotherapy (FOLFOXIRI) plus anti‐EGFR antibody (cetuximab or panitumumab) as first-line treatment were included [15]. Table 1 describes the characteristics of the eligible studies and treatment schemes in more detail.

**Table 1:** Study characteristics

First author	Number of patients	Age, median years (range)	Target population	Schemes	Doses	Cycle
Hu et al. (2021) [15]	67	52 (28–70)	BRAF/RAS wild type initially unresectable liver-limited mCRC	mFOLFOXIRI + cetuximab	Cetuximab 500 mg/m^2^, oxaliplatin 85 mg/m^2^, irinotecan 165 mg/m^2^, folinic acid 400 mg/m^2^, 5-FU 2800 mg/m^2^ for 46 h every 2 weeks	7 (4–12)
	34	55 (29–70)		mFOLFOXIRI	Oxaliplatin 85 mg/m^2^, irinotecan 165 mg/m^2^, folinic acid 400 mg/m^2^, 5-FU 2800 mg/m^2^ for 46 h every 2 weeks	6 (2–12)
Modest et al. (2019) [16]	63	58 (31–76)	RAS wild-type untreated mCRC	mFOLFOXIRI + panitumumab	Panitumumab 6 mg/kg, oxaliplatin 85 mg/m^2^, irinotecan 150 mg/m^2^, folinic acid 200 mg/m^2^, 5-FU 3000 mg/m^2^ for 46 h every 2 weeks	11 (2–12)
	33	60 (32–77)		m FOLFOXIRI	Oxaliplatin 85 mg/m^2^, irinotecan 150 mg/m^2^, folinic acid 200 mg/m^2^, 5-FU 3000 mg/m^2^ for 46 h every 2 weeks	11 (2–12)
Assenat et al. (2011) [11]	42	60 (32–76)	Unresectable mCRC	FOLFIRINOX + cetuximab	Cetuximab 400 mg/m^2^, oxaliplatin 85 mg/m^2^, irinotecan 180 mg/m^2^, 5-FU 400 g/m^2^, 5-FU 2800 mg/m^2^ for 46 h every 2 weeks	9 (1–12)
Fornaro et al. (2013) [14]	37	63 (33–72)	Quadruple wild-type (KRAS, NRAS, HRAS, BRAF) mCRC	A slightly modified GONO-FOLFOXIRI + panitumumab	Panitumumab 6 mg/kg, oxaliplatin 85 mg/m^2^, irinotecan 150 mg/m^2^, folinic acid 200 mg/m^2^, 5-FU 2400 mg/m^2^ for 46 h every 2 weeks	11 (3–16)
Garufi et al. (2010) [12]	43	61 (33–75)	Unresectable liver-limited mCRC	Chronomodulated FOLFOXIRI + cetuximab	Cetuximab 400 mg/m^2^/2 weeks—250 mg/m^2^ weekly; irinotecan 110–130 mg/m^2^, oxaliplatin 15–20 mg/m^2^/day × 4 days, folinic acid 150 mg/m^2^/day × 4 days, 5-FU 550–600 mg/m^2^/d × 4 days every 2 weeks	6 (3–15)
Saridaki et al. (2012) [13]	30	64 (36–70)	KRAS wild-type mCRC	FOLFOXIRI + cetuximab	Cetuximab 500 mg/m^2^, oxaliplatin 65 mg/m^2^, irinotecan 150 mg/m^2^, folinic acid 200 mg/m^2^, 5-FU 400 g/m^2^, 5-FU 1200 mg/m^2^ for 44 h every 2 weeks	/

*mCRC* Metastatic colorectal cancer, *5-FU* 5-fluorouracil

The specific main outcome of this meta-analysis is the ORR and R0 resection rate, with secondary outcomes being adverse events during treatment. By introducing random effects, there can be a certain correlation between individual observations, so it can be used to fit nonindependent observation data.

### ORR

**Fig. 2:**
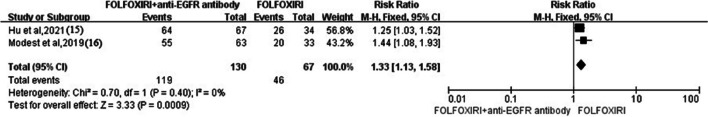
Forest plot. The overall response rate in patients who received FOLFOXIRI + anti-EGFR antibody or FOLFOXIRI

**Fig. 3:**
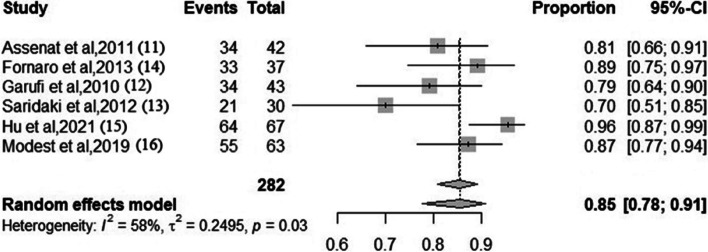
Forest plots. The pooled proportion of overall response rate in patients who received FOLFOXIRI + anti-EGFR antibody

Two studies reported the ORR (complete and partial responses) between FOLFOXIRI + anti-EGFR antibody arm and FOLFOXIRI arm [17]. The data showed a significant benefit for the FOLFOXIRI + anti-EGFR antibody arm (*RR* 1.33; 95% *CI*, 1.13–1.58; *I*^2^ = 0% according to the fixed-effects mode, *P* < 0.05). The results are shown in Fig. 2. All six studies presented ORR data in patients who received FOLFOXIRI + anti-EGFR antibody. The pooled ORR was 85% (95% *CI*, 0.78–0.91; *I*^2^ = 58% according to the random-effects model). The results are shown in Fig. 3.

### The rate of R0 resection

**Fig. 4:**
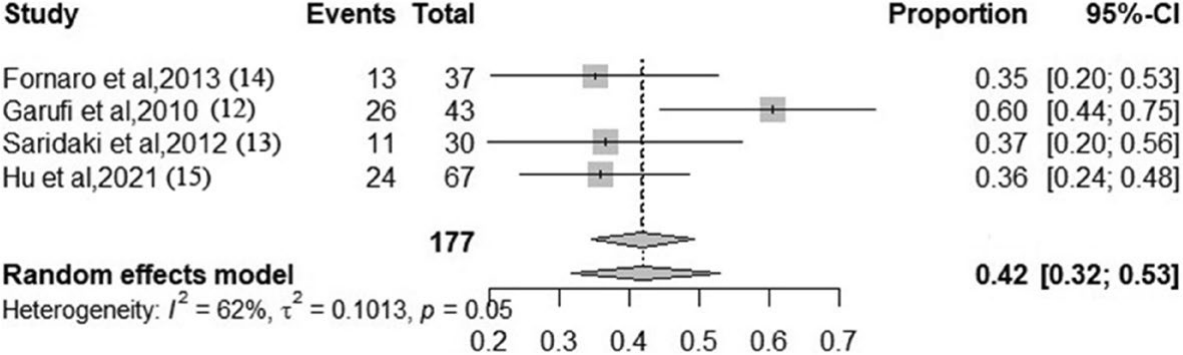
Forest plots. The pooled proportion of R0 resection rate in patients who received FOLFOXIRI + anti-EGFR antibody

Four studies reported the rate of R0 resection data in patients who receiving FOLFOXIRI + anti-EGFR antibody. The pooled rate of R0 resection was 42% (95% *CI*, 0.32–0.53; *I*^2^ = 62% according to the random-effects model). The results are shown in Fig. 4.

### Outcome (median OS and PFS)

The range of median PFS between all the six studies were 9.5–15.5 months, with weighted pooled median PFS mean 11.7 months. Median OS in the original data was not reached in two studies. The remaining four studies reported that the range of median OS was 24.7–37 months, with weighted pooled median OS mean 31.9 months. More detailed outcome data are shown in Table 2.

**Table 2:** Detailed outcome data

First author	Schemes	ORR	mPFS (months)	mOS (months)
Hu et al. (2021) [15]	mFOLFOXIRI + cetuximab	95.5%	15.5	Not reached
	mFOLFOXIRI	76.5%	14.2	33.2
Modest et al. (2019) [16]	mFOLFOXIRI + panitumumab	87.3%	9.7	35.7
	m FOLFOXIRI	60.6%	4	29.8
Assenat et al. (2011) [11]	FOLFIRINOX + cetuximab	80.9%	9.5	24.7
Fornaro et al. (2013) [14]	A slightly modified GONO-FOLFOXIRI + panitumumab	89%	11.3	Not reached
Garufi et al. (2010) [12]	Chronomodulated FOLFOXIRI + cetuximab	79.1%	14	37
Saridaki et al. (2012) [13]	FOLFOXIRI + cetuximab	70%	10.2	30.3

### Adverse events

**Fig. 5:**
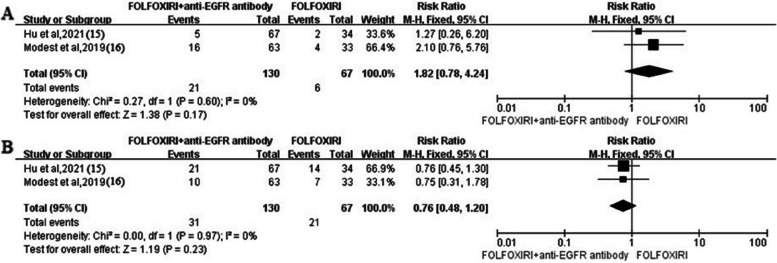
Forest plots. The reported grades 3 or 4 adverse event rates in patients who received FOLFOXIRI + anti-EGFR antibody or FOLFOXIRI (**A** diarrhea. **B** Neutropenia)

**Fig. 6:**
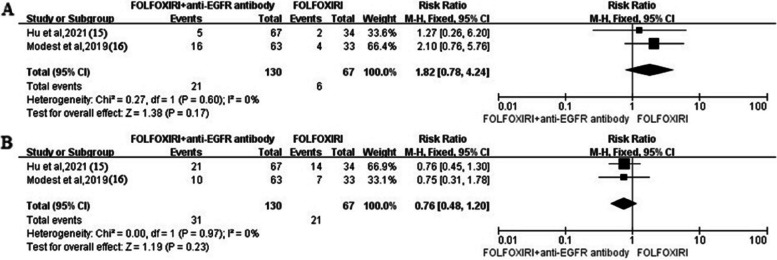
Forest plots. The pooled proportion of the reported grades 3 or 4 adverse event rates in patients who received FOLFOXIRI + anti-EGFR antibody (**A** diarrhea. **B** neutropenia)

Diarrhea, neutropenia, fatigue, acneiform exanthema/rash, stomatitis, and so on were the common grades 3 and 4 adverse events, especially diarrhea and neutropenia. Two studies reported the grades 3 and 4 diarrhea and neutropenia between FOLFOXIRI + anti-EGFR antibody arm and FOLFOXIRI arm. FOLFOXIRI + anti-EGFR antibody arm seemingly showed a higher risk of diarrhea (*RR* 1.82, 95% *CI* 0.78–4.24, *I*^2^ = 0%) and a lower risk of neutropenia (*RR* 0.76, 95% *CI* 0.48–1.2, *I*^2^ = 0%), but no significant difference between two arms (*P* > 0.05) (Fig. 5). The pooled event rates per 100 patients for grades 3 or 4 adverse events are presented in forest plots in 5 studies (Fig. 6). The pooled rates per 100 patients were 31 (95% *CI*: 16–51, *I*^2^ = 86%) for diarrhea and 30 (95% *CI*: 21–41, *I*^2^ = 70%) for neutropenia.

## Discussion

The results of this meta-analysis confirm the feasible and effective strategy with intensification triplet chemotherapy plus anti-EGFR antibody in patients with initially unresectable metastatic colorectal cancer. We show that FOLFOXIRI + cetuximab/panitumumab resulted in a particularly high ORR of 85%, which translated into a high secondary R0 resection rate of 42% in a population initially unresectable mCRC.

Surgical resection was the only potential cure strategy for mCRC, especially in limited liver metastases (CLM). There is a clear need for an effective schedule that yields high objective response rates leading to secondary resection [18]. Due to intensification strategy and multidisciplinary teamwork, patients might benefit more from this schedule that aims to achieving complete removal of all tumor masses and no evidence of disease (NED) [19]. In previous studies, it rarely reported such high ORR and R0 resection rate in unresectable metastatic colorectal cancer. For example, a systematic review and pooled analysis reported the ORR of 69% and R0 resection rate of 28% in overall population of initially unresectable mCRC treated with FOLFOXIRI plus bevacizumab [20].

In some recent phase III trials, it was reported that a significant improvement in ORR might translate into OS benefit in anti-EGFR-containing regimens [21]. In our meta-analysis, the pooled median DFS and OS of patients with combined treatment were 11.7 months and 31.9 months, respectively. One of those trials (FOCULM trial) about mFOLFOXIRI with or without cetuximab as conversion therapy in patients with RAS/BRAF wild-type unresectable liver metastases colorectal cancer showed the median OS of 33.2 months in the control group (mFOLFOXIRI only). In addition, two trials included showed that median OS was not reached in the follow-up time. We conjecture daringly that the median OS in mFOLFOXIRI with cetuximab group may be reasonably better. FOCULM trial also indicated possibly improved median PFS of cetuximab plus mFOLFOXIRI, but the VOLFI trial demonstrated no apparent difference in PFS.

No unexpected toxicities shown in FOLFOXIRI combine with panitumumab or cetuximab regimens in our meta-analysis. Triplet-drug chemotherapy combined with anti-EGFR antibody scheme seems not to increase toxicity (diarrhea or neutropenia) significantly than triplet-drug chemotherapy. The main concerned grades 3 or 4 adverse events of the regimens were diarrhea, neutropenia, fatigue, acneiform exanthema/rash, stomatitis, and so on, especially diarrhea (31%) and neutropenia (30%). In some trials, the incidence of adverse event may be reduced by reducing the dosage of drugs such as irinotecan and receiving some prevention measures such as granulocyte-colony-stimulating factor and early supportive treatment.

There were some limitations in this study. Firstly, due to no results of phase III clinical trial reported in these field, we took in both phase II studies involving single-arm trials and two randomized trials. The population sample size was too small to research deeply. Secondly, there are some subtle differences in the baseline characteristics. All the patients involved in those trials are unresectable initially. In most trials, they may have a chance of secondary resection with curative intent, especially those trials involved in patients with liver-limited metastases. But in one trial, almost all of patients with significant tumor load were definitively inoperable or unresectable initially. Thirdly, we did not conduct subgroup analysis about RAS/BRAF state. The treatment activity and long-term survival for RAS/BRAF mutant-type patients usually are lowed. So our study may hide more promising results. On the other hand, RAS/BRAF mutant-type patients with limited treatment might benefit more from the intensification triplet chemotherapy plus anti-EGFR antibody scheme.

In conclusion, our findings show that triplet-drug chemotherapy (FOLFOXIRI) combined with anti-EGFR antibody (panitumumab or cetuximab) represents a very effective therapeutic combination associated with a significant ORR and R0 rection rate for patients with molecularly unselected and surgically unresectable metastatic CRC. The intensification triplet chemotherapy plus anti-EGFR antibody scheme was feasible and effective in patients with initially unresectable metastatic colorectal cancer. Patients might benefit more from this conversion schedules that are achieving high ORR and R0 resection rate. It might bring in survival benefit. Several phase III trials are currently under way. We are expected to be further validated in the large sample phase III randomized comparison clinical trials in the future [22–25].

## Data Availability

The figures and tables used to support the findings of this study are included in the article.
